# A comparison of approaches to measuring maternal mortality in Bangladesh, Mozambique, and Bolivia

**DOI:** 10.1186/s12963-022-00281-8

**Published:** 2022-01-15

**Authors:** Kavita Singh, Qingfeng Li, Karar Zunaid Ahsan, Sian Curtis, William Weiss

**Affiliations:** 1 Department of Maternal and Child Health, Gillings School of Global Public Health, University of North Carolina at Chapel Hill, Chapel Hill, NC USA; 2 Carolina Population Center, University of North Carolina at Chapel Hill, Chapel Hill, NC USA; 3 Department of International Health, Bloomberg School of Public Health, Johns Hopkins University, Baltimore, MD USA; 4 Public Health Institute, Oakland, CA USA

**Keywords:** Maternal mortality, Pregnancy-related mortality, Population census, Sample survey, Vital registration, Bangladesh, Bolivia, Mozambique

## Abstract

**Background:**

Many low- and middle-income countries cannot measure maternal mortality to monitor progress against global and country-specific targets. While the ultimate goal for these countries is to have complete civil registrations systems, other interim strategies are needed to provide timely estimates of maternal mortality.

**Objective:**

The objective is to inform on potential options for measuring maternal mortality.

**Methods:**

This paper uses a case study approach to compare methodologies and estimates of pregnancy-related mortality ratio (PRMR)/maternal mortality ratio (MMR) obtained from four different data sources from similar time periods in Bangladesh, Mozambique, and Bolivia—national population census; post-census mortality survey; household sample survey; and sample vital registration system (SVRS).

**Results:**

For Bangladesh, PRMR from the 2011 census falls closely in line with the 2010 household survey and SVRS estimates, while SVRS’ MMR estimates are closer to the PRMR estimates obtained from the household survey. Mozambique's PRMR from household survey method is comparable and shows an upward trend between 1994 and 2011, whereas the post-census mortality survey estimated a higher MMR for 2007. Bolivia's DHS and post-census mortality survey also estimated comparable MMR during 1998–2003.

**Conclusions:**

Overall all these data sources presented in this paper have provided valuable information on maternal mortality in Bangladesh, Mozambique, and Bolivia. It also outlines recommendations to estimate maternal mortality based on the advantages and disadvantages of several approaches.

**Contribution:**

Recommendations in this paper can help health administrators and policy planners in prioritizing investment for collecting reliable and contemporaneous estimates of maternal mortality while progressing toward a complete civil registration system.

**Supplementary Information:**

The online version contains supplementary material available at 10.1186/s12963-022-00281-8.

## Introduction

Maternal mortality is difficult to measure in many low- and middle-income countries, where 99% of global maternal deaths occur. In these settings, maternal deaths are frequently not captured in either a country’s routine health information system (HIS) or the civil registration system. HIS maternal mortality data can yield valuable information, but may not be generalizable, to the larger population in settings where many deliveries and maternal deaths occur in non-facility settings. Civil registration is a government system of reporting vital events including births, deaths, and marriages. Out of 193 countries, only 63 countries have civil registration data characterized as complete with good attribution of cause of death, which would allow for the calculation of measures of maternal mortality [35, 37, 38].

Accurate estimates of maternal mortality are needed to monitor progress against programs and initiatives. United Nations’ Millennium Development Goal (MDG) 5, a three-quarters reduction in maternal mortality from 1990 to 2015, was the most challenging and least met of the health-related MDGs [32]. Getting data to monitor progress against this goal was a considerable challenge for many countries. The global focus on maternal mortality reduction has not diminished in the post-MDG era. Though the Sustainable Development Goal (SDG) 3 is broadly focused on improving health for all, target 1 is specific to maternal health. The target is to reduce the global maternal mortality ratio (MMR) to less than 70 maternal deaths per 100,000 live births by 2030, with no individual country exceeding an MMR of 140 maternal deaths per 100,000 live births [12]. In order to measure progress against this target, accurate and timely data on maternal mortality is needed. Ultimately the goal is for countries to have complete civil registrations systems in which all vital events, including births and maternal deaths, would be documented. As low- and middle-income countries work to strengthen their civil registration systems, other interim strategies are needed that would provide timely estimates of maternal mortality.

In this paper, we use a case study approach to compare and contrast methodologies and estimates obtained from three different maternal mortality data sources from similar time periods in Bangladesh, Mozambique, and Bolivia. These three countries were chosen for this analysis because they had the data needed for the comparisons and attained a greater reduction in maternal mortality than the respective regional averages during the MDG period [2]. The United Nations Maternal Mortality Estimation Inter-agency Group (MMEIG) periodically generate internationally comparable modeled MMR estimates available country data and multilevel regression model for countries without sufficient high-quality information from vital registration systems [4]. Based on MMEIG estimates, maternal mortality in Bangladesh fell from 569 maternal deaths per 100,000 live births in 1990 to 176 maternal deaths per 100,000 live births in 2015 [38]. Bangladesh fell just short of meeting MDG 5 with a 69% reduction in maternal mortality, and the current ratio stands at 173 maternal deaths per 100,000 live births [12]. In Mozambique, there was a 65% reduction in the MMR from 1390 maternal deaths per 100,000 live births in 1990 to 489 maternal deaths per 100,000 live births [38]. Mozambique has achieved further reductions in maternal mortality, which now stands at 289 maternal deaths per 100,000 live births [12]. Bolivia achieved a 52% reduction in maternal mortality with a decline from 425 maternal deaths per 100,000 live births in 1990 to 206 maternal deaths per 100,000 live births in 2015 [38]. Maternal mortality in Bolivia currently is 155 maternal deaths per 100,000 live births [12].

Maternal mortality is often considered as a litmus test of the status of women and the adequacy of the overall healthcare system in a country [34], and MMR is a core indicator for sexual and reproductive health within the global developmental agenda [11]. However, MMR estimates and accurate identification of the causes of maternal death are still a complex and difficult challenge. A recent review noted the need for low- and middle-income countries to have guidance on which methods of estimating maternal mortality would be most feasible for their setting [21]. Thus, the objective of this paper is to inform low- and middle-income countries on potential options for measuring maternal mortality from different data sources. We also discuss the advantages and disadvantages of each approach as well as the potential ways to strengthen each approach.

## Data/methods

The data sources by each of the three countries can be categorized as follows: (1) national population census; (2) census followed by a mortality survey; (3) household sample survey; and (4) sample vital registration system (SVRS). A detailed description of the technical approaches and a table of the advantages and disadvantages of each approach is included in Additional file 1. This section gives an overview of each of the approaches.

For Bangladesh, we compare three data sources and methods for measuring maternal mortality. They are: (1) the 2011 National Population and Housing Census [6],(2) the 2010 Bangladesh Maternal Mortality Survey (BMMS), a large-scale household sample survey and follow-up verbal autopsy [26],and (3) SVRS data from 2006 to 2011 [7]. In Mozambique and Bolivia, the data sources and methods compared are similar. In both countries a post-census mortality survey was conducted after a census, and the Demographic and Health Surveys (DHS) in each country includes estimates of maternal mortality. Each of the data sources allows for the calculation of either pregnancy-related mortality ratio (PRMR) or MMR. Pregnancy-related mortality is defined as the death of a woman during pregnancy or within 42 days of pregnancy [36]. In contrast, maternal mortality is defined as the death of a woman while pregnant or within 42 days of the termination of pregnancy, irrespective of the duration and the site of the pregnancy, from any cause related to or aggravated by the pregnancy or its management but not from accidental or incidental causes [36]. A key difference between these two measures is the availability of cause of death information. To measure maternal mortality, both the timing of the death in relation to a pregnancy and the cause of death need to be known. With pregnancy-related mortality, only timing of the death in relation to a pregnancy needs to be known.

Each of the aforementioned sources are described below, and Table 1 provides a summary of the data sources available in each country and indicates whether the measure is pregnancy-related mortality or maternal mortality.

**Table 1:** Summary of data sources

Data source	Years of data collection	Coverage	Organization responsible for data collection	Measure(s)	Method
Bangladesh Population and Housing Census	2011	168,000 households	Bangladesh Bureau of Statistics (BBS)	Pregnancy-related mortality	Census questions
Bangladesh Maternal Mortality Survey	2010	175,000 households	National Institute of Population Research and Training (NIPORT), with technical assistance from MEASURE Evaluation, the International Centre for Diarrhoeal Disease Research, Bangladesh (icddr,b), and U.S. Agency for International Development/Bangladesh	Pregnancy-related mortality and maternal mortality	(a) Direct sisterhood (b) Household deaths (c) Household deaths and verbal autopsy
Bangladesh Sample Vital Registration System	2006‒2011 (continuous)	206,522 households	Bangladesh Bureau of Statistics (BBS)	Maternal mortality	Death Schedule
Mozambique Census and Post-census mortality survey (Mozambique Inquerito Sobre Causas de Mortalidade—INCAM)	2007‒2008	33,290 households (census) 1643 female deaths age 15‒49 years	Mozambique National Institute of Statistics, U.S. Census Bureau, MEASURE Evaluation, U.S. Centers for Disease Control and Prevention	Maternal mortality	Census questions and post-census verbal autopsy
Mozambique Demographic and Health Survey	2003 and 2011	12,315 households (2003) 13,919 households (2011)	Mozambique National Institute of Statistics, Mozambique Ministry of Health and MEASURE DHS+/ORC Macro	Pregnancy-related mortality	Indirect sisterhood method
Bolivia Census and Post-census Maternal Mortality Survey	2001 and 2002	3362 pregnancy-related deaths (census)	Bolivian National Institute of Statistics, Bolivian Ministry of Planning and Coordination	Pregnancy-related mortality	Census questions and post-census verbal autopsy
Bolivia Demographic and Health Survey	2003	19,942 households	Bolivian Ministry of Health and Sports, U.S. Agency for International Development, Canada Health Fund, World Bank, United Nations Population Fund, United Nations Children's Fund, World Food Program, MEASURE DHS+/ORC Macro	Maternal mortality	Indirect sisterhood method

### Census

In order to estimate maternal or pregnancy-related mortality from a census, only a few specific data elements are needed. The reference period for these data elements is typically the past year.

- Population size by five-year age group and sex
- Deaths by five-year age group and sex
- Deaths to women who are pregnant, in labor/delivery and in the six weeks/two months postpartum
- Births to women by five-year age group
- Number of children ever born to women by five-year age group.

The Fifth Population and Housing Census of Bangladesh in 2011 included a short questionnaire to collect information on population by age and sex, but not on mortality or fertility. Following the recommendations of the United Nations Statistics Division [33], Bangladesh Bureau of Statistics (BBS) conducted the 2011 Population and Housing Census in three phases: basic data about all households and individual members of the households were collected in phase I,quality and coverage of the main census count were verified through a Post-Enumeration Check (PEC) survey in phase II; and detailed socio-economic and demographic (including fertility and mortality) information was collected in a sample survey of 168,000 households following the census to supplement the main census estimates in phase III [8].

### Post-census mortality survey

Following the Third General Population and Housing Census in Mozambique in 2007 [20], a post-census mortality survey was conducted. A total of 10,080 deaths including 213 maternal deaths were included in the survey [25]. This method yielded estimates of maternal mortality because of the available of cause of death information from the verbal autopsy questionnaires. Likewise, Bolivia conducted a post-census survey followed their 2001 census. This survey covered all 1504 households that had reported a pregnancy-related death in the 2001 census [19].

### Household survey

The BMMS 2010 was a large-scale, nationally representative sample survey which included 175,000 households. Fieldwork was carried out between January and August 2010. This survey employed several methods to obtain maternal mortality estimates—the direct sisterhood method, questions on household deaths and a verbal autopsy [26]. The direct sisterhood method provides pregnancy-related mortality estimates for the time periods 1996‒2000, 2001‒2005, 2006‒2010 and for the period 2008‒2010. Each household was also asked if any death occurred since October 2006. If yes, their name, sex, and age at death were recorded. For deaths of women age 13‒49 years, additional questions were asked to ascertain whether she was pregnant, in labor/delivery or within two months of delivery at the time of death. For all household deaths of women age 13‒49 years, a follow-up verbal autopsy was conducted. Maternal deaths were identified on basis of review by two (or three, in case of disagreement between the first two physicians) physicians using the International Classification of Disease Revision 10 [36]. The household deaths methods (with and without the verbal autopsy) provide maternal mortality estimates for the period from early 2007 to early 2010.

The Mozambique 2011 DHS, and the Bolivia 2003 DHS, employed the indirect sisterhood method to obtain estimates of pregnancy-related mortality. With the indirect sisterhood method, women are asked about the survival status of their sisters. For any sister who died, questions are asked on whether she was pregnant around the time of death. The questions for the indirect sisterhood method are relatively easy to incorporate in a survey, but because of assumptions with the methodology, it should not be used in settings with low fertility or with recent large declines in fertility [1, 14]. The time reference for the indirect sisterhood method is generally 7–12 years before the survey. The reference period of Mozambique post-census mortality survey was June 2006–June 2007, which was in the middle of the Mozambique 2011 DHS reference period. As a result, the estimates of the two sources are comparable. The estimated from the Bolivia 2003 would fall earlier than the 2001 Bolivia post-census maternal mortality estimates but are still fairly close in time.

### Sample vital registration system (SVRS)

Bangladesh’s SVRS involves continuous data collection on vital events (births, deaths, marriages), migration, disability and key demographic variables. The SVRS began in 1980 and is overseen by the country’s national statistical office BBS. Initially 103 primary sample units (PSU), each comprising about 250 households, were followed over time but this increased to 1000 primary sample units by 2002. The 1000 PSUs include approximately 206,522 sample households [7]. The sample is nationally representative. Vital events are captured by a dual recording system as outlined by Chandrasekaran and Deming [9, 10], which used for estimating the completeness of a data source by cross-matching its records case by case against those of another source. Births to women of all ages are captured. On the death schedule there are eight questions regarding the individual who died including a question on cause of death. These questions are asked by the local registrar or statistics official, and annual reports on the SVRS include a note of caution about the accuracy of the cause of death data because it was not collected by clinicians. In addition the list of maternal causes is not exhaustive. There are six codes for maternal deaths which are listed below:

- Pregnancy complication/loss of thirst/loss of appetite/edema of feet
- Delivery complication/retained placenta/acute pain during delivery/uterine rupture
- Postpartum hemorrhage
- Abortion complications
- Antepartum hemorrhage
- Excess vaginal discharge (*sutika*, indicating postpartum infection).

For the purpose of this activity, we will use the maternal mortality estimates provided from the SVRS from 2006 to 2011 to be comparable to the 2011 census. The SVRS presents MMRs for women of all ages.

## Results

### Bangladesh

#### Census

**Fig. 1:**
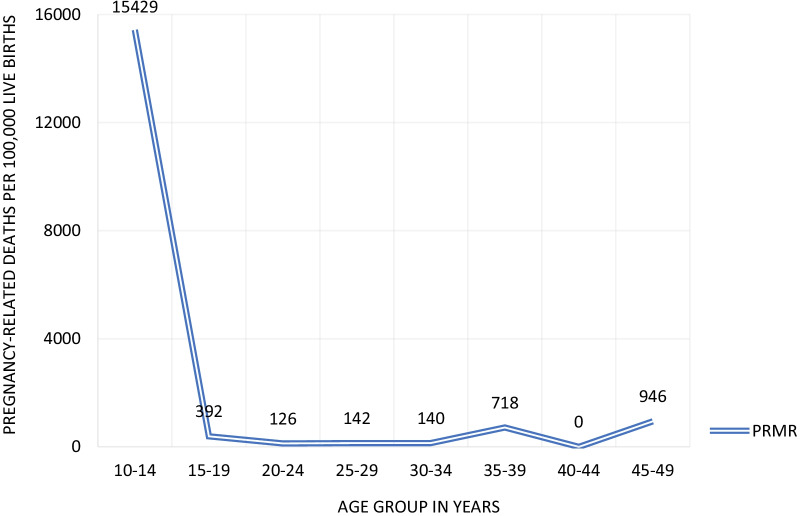
Pregnancy-related mortality ratio by age groups estimated form population census, Bangladesh 2011. *Data Source*: BBS [6]

The Socio-Economic and Demographic Report of the 2011 Census presents the PRMRs for the period October 2010‒October 2011 by five-year age groups from the 2011 population census (Fig. 1). After accounting for the sampling design, BBS estimated a total number of 2,486,306 live births to women age 10‒49 years within 12 months prior to the sample survey; and that there were 5411 pregnancy-related deaths. That gives an estimated pregnancy related mortality ratio of 218 per 100,000 live births for women age 10‒49 the time period 2010‒2011. The PRMR estimate is 206 for women age 15‒49 years for the time period of 2010‒2011.

Fertility in the population census, estimated from the sample survey, might be underestimated. The estimated total fertility rate (defined as the number of children born to a woman if she were to pass through her childbearing years according to a current set of age specific fertility rates) of 1.90 children per woman is lower than the estimate of 2.32 from Bangladesh DHS conducted around the same time (July–December 2011). Since the 2001 population census did not include fertility questions, we could only apply the single-census version of the P/F (parity to fertility) ratio method to adjust the fertility. The method adjusted the total fertility rate upward to 2.77 children per woman, which in return results in a reduction of PRMR to 141. The adjusted result needs to be interpreted with caution for two reasons. First, the application of the single-census version of the P/F method is only appropriate in a context of constant fertility over an extended period of time, which is not the case for Bangladesh. Second, like in most censuses, deaths are also likely to be underreported. Missing mortality data from the 2001 census makes it impossible to adjust for the mortality underreporting. As a result, upward adjusting fertility without adjusting for the potential underreporting in deaths may lead to an overestimation of the PRMR. The PRMR adjusted only for fertility is presented mainly to present an alternative measure.

#### Household survey

**Fig. 2:**
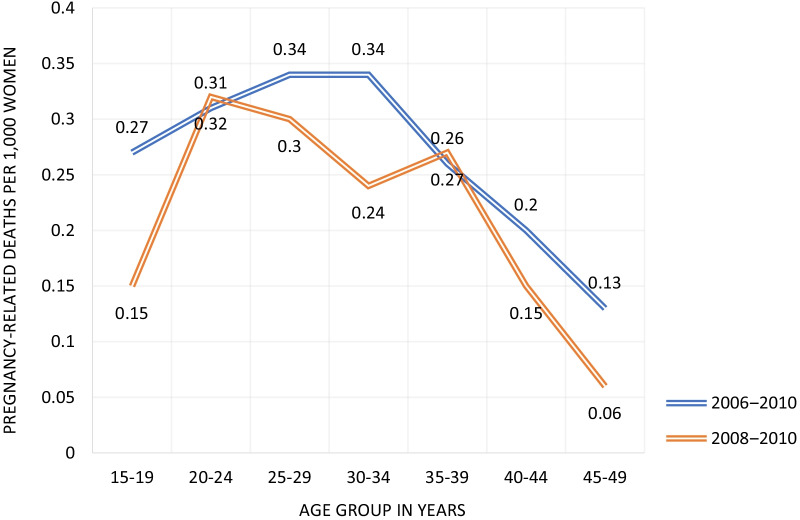
Pregnancy-related mortality rate by age groups estimated by sisterhood method, Bangladesh. *Data Source*: NIPORT et al. [26]

With the direct sisterhood method employed in the BMMS 2010, the number of pregnancy-related deaths among the sampled respondents’ sisters was divided by sister exposure time (sister*years) to obtain pregnancy-related maternal mortality rates. This is often done by 5 year age groups for the sister’s age at death. In order to obtain estimates of PRMRs, the overall pregnancy-related maternal mortality rate can be divided by the general fertility rate, which is calculated as the number of live births per women age 15‒49 years. Though this method yields results for time periods as early as 1996‒2001, we only present the more recent estimates for the periods 2006‒2010 and 2008‒2010. For the period 2006‒2010, the PRMR estimate is 301 maternal deaths per 100,000 live births, and for the period 2008‒2010 the estimate is 257 maternal deaths per 100,000 live births. The pregnancy-related mortality rate by age groups estimated by sisterhood methods are shown in Fig. 2.

**Fig. 3:**
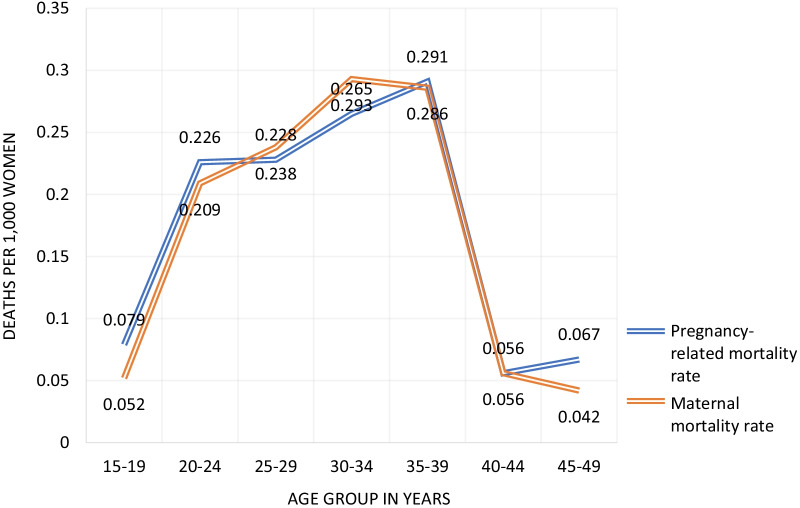
Pregnancy-related mortality rate and maternal mortality rate by age groups estimated from verbal autopsy data, Bangladesh. *Data Source*: NIPORT et al. [26]

In the BMMS, a verbal autopsy module was also administered—respondents were asked about household deaths occurring from October 2006 to the time of the survey. Both pregnancy-related and maternal mortality estimates from this method refer to a period from about early 2007 to early 2010. For households where a death to a women between 15 and 49 occurred, a follow-up verbal autopsy was conducted. Deaths to women can be categorized into 5 year age groups. Using the household deaths data alone (without the verbal autopsy data), pregnancy-related deaths are divided by exposure time (woman-years) to obtain pregnancy-related mortality rates. These mortality rates can then be divided by age-specific fertility rates to obtain age specific pregnancy-related morality ratios. The overall pregnancy-related mortality ratio is obtained by dividing the overall pregnancy-related mortality rate by the general fertility rate. This value is 201 pregnancy-related deaths per 100,000 live births. Using data from the verbal autopsy, it is possible to classify deaths as maternal deaths because of the cause of death information. Maternal deaths can be categorized into five year age groups. Maternal deaths are divided by woman-years exposure to obtain maternal mortality rates. These rates are then divided by age specific fertility rates to obtain age-specific MMRs. The overall MMR is obtained by dividing total maternal deaths by woman-years exposure and then dividing by the general fertility rate. The overall MMR is 194 maternal deaths per 100,000 live births. Results for both the pregnancy-related mortality rate and maternal mortality rate by age groups estimated by verbal autopsy are shown in Fig. 3.

#### SVRS

**Fig. 4:**
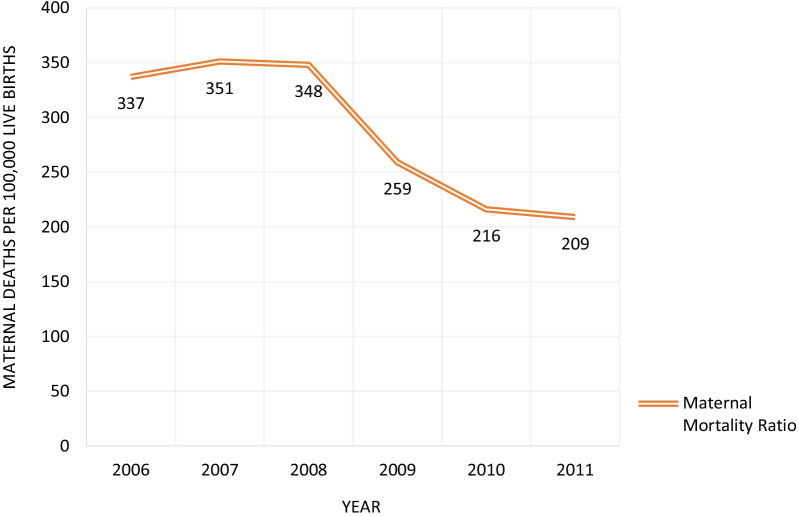
Trends in maternal mortality ratio from the sample vital registration system, Bangladesh 2006‒2011. *Data Source*: BBS [7]

The SVRS provides both the numerators (maternal deaths) and denominators (live births) to calculate MMRs. Figure 4 presents the trends in MMR during 2006‒2011. The values range from 351 maternal deaths per 100,000 live births in 2007 to 209 maternal deaths per 1000 live births in 2011. There is a particularly large decline from 2008 to 2009 when the MMRs drops from 348 to 259 maternal deaths per 100,000 live births.

Table 2 summarizes the estimates obtained from the three different sources of pregnancy related and/or maternal mortality data for Bangladesh. The 2011 Population Census allows for the calculation of pregnancy-related mortality only. The BMMS 2010 included questions for three different methods of estimating maternal mortality. Two of the methods give estimates of pregnancy-related mortality (household deaths and the sisterhood method). The third method allows for the calculation of maternal mortality estimates by combining data on household deaths from the survey with cause of death information from the verbal autopsy. The SVRS provides estimates for maternal mortality because information on cause of death was obtained; however, a caveat is that this information was obtained from lay people and not clinicians. Since the estimates for the census and the BMMS 2010 reflect at least a period of two years, averages were taken from the annual SVRS estimates for comparative purposes. Overall, the estimates appear to be fairly comparable to one another. The unadjusted PRMR of 206 maternal deaths per 100,000 live births from the census (for the time period 2010‒2011) falls closely in line with the BMMS 2010 estimates from the household method. These estimates were 201 pregnany-related deaths per 100,000 live births for the PRMR and 194 maternal deaths per 100,000 live births for the MMR for the period 2007‒2010. The average pregnancy-related estimate from the SVRS for 2010‒2011 was 212 maternal deaths per 100,000 live births. Comparing the SVRS and BMMS 2010 estimates for the 2006‒2010, 2007‒2010 and 2008‒2010 time periods, it appears that the SVRS estimates are closer to the estimates obtained from the direct sisterhood method rather than the verbal autopsy method in household survey that estimates the MMR.

**Table 2:** Comparison of estimates of pregnancy-related and maternal mortality ratios from the 2011 population census, Bangladesh maternal mortality survey 2010 and sample vital registration system 2006‒2011

Year	Estimate (per 100,000 live births)	Measure	Source
2006	337	Maternal mortality ratio	Sample Vital Registration System
2006‒2010	301	Pregnancy-related mortality ratio	Bangladesh Maternal Mortality Survey 2010 (Sisterhood)
2006‒2010 (average)	302	Pregnancy-related mortality ratio	Sample Vital Registration System (average from 2006‒2010)
2007	351	Maternal mortality ratio	Sample Vital Registration System
2007‒2010	201	Pregnancy-related mortality ratio	Bangladesh Maternal Mortality Survey 2010 (Household)
2007‒2010	194	Maternal mortality ratio	Bangladesh Maternal Mortality Survey 2010 (Household and Verbal Autopsy)
2007‒2010 (average)	293	Pregnancy-related mortality ratio	Sample Vital Registration System (average from 2007‒2010)
2008	348	Maternal mortality ratio	Sample Vital Registration System
2008‒2010	257	Pregnancy-related mortality ratio	Bangladesh Maternal Mortality Survey 2010 (Sisterhood)
2008‒2010 (average)	275	Pregnancy-related mortality ratio	Sample Vital Registration System (average from 2008‒2010)
2009	259	Maternal mortality ratio	Sample Vital Registration System
2010	216	Maternal mortality ratio	Sample Vital Registration system
2010‒2011	206	Pregnancy-related mortality ratio	Population Census 2011
2010‒2011 (average)	212	Pregnancy-related mortality ratio	Sample Vital Registration System (average from 2010‒2011)
2011	209	Maternal mortality ratio	Sample Vital Registration System

*Data Source*: BBS [6], BBS [7], NIPORT et al. [27]

### Mozambique

#### Post-census mortality survey

Mozambique conducted a Population and Housing Census from 1 to 15 August 2007. For all female deaths age 12‒50 years, the census questionnaire asked if the death occurred during pregnancy, delivery, or two months after the end of pregnancy. A post-census mortality survey called Mozambique Inquerito Sobre Causas de Mortalidade (INCAM) was implemented to follow up on deaths reported in the census. It used a cluster sample of 4.5% of all deaths reported in the census. The aim was to collect additional information on deaths reported in the census and to administer a verbal autopsy to identify the cause of death.

The mortality-related information collected in the two sources allowed the estimates of pregnancy-related mortality in the census and INCAM. Due to verbal autopsy, INCAM is believed to be more accurate than the census in reporting deaths and determining maternal deaths. Within the INCAM sample areas, the census recorded a total of 18,175 deaths. Verbal autopsy interviewers returned to the households reporting those deaths. The INCAM interviews determined that 6353 or more than a third of those reported deaths actually out of the scope. 4891 of those out of scope deaths occurred outside of the census reference period August 1, 2006–July 31, 2007. Other out of scope deaths included those not residing within the INCAM areas, duplicated reporting, and stillbirths. A total of 1562 deaths reported in the census could not be located for reasons like dissolved households. Sixty-two household declined to participate in the INCAM interviews. INCAM team identified 185 deaths that were missed in the census.

The ratio of estimates of pregnancy-related deaths to all female deaths age 15‒49 years from the two sources is 2.36, which is quite large, considering the relatively short gap between the two data collections. See Table 3. The inclusion of verbal autopsy in INCAM is apparently the major reason for the large ratio. Another possible reason is the varying gap between census and INCAM fieldwork at the provincial level. The longer the gap, the more difficult it will be to locate households and follow up on the reported deaths. The province of Maputo started INCAM fieldwork in October 2007, 2 months after the census. The province of Tete finished the fieldwork in May 2008, 9 months after the census. However, the impact of the varying gap may not be substantial since the percentage of follow-up interviews that could be realized was fairly high at 84%.

**Table 3:** All-cause and pregnancy-related deaths in the INCAM sample areas

Deaths	Census	INCAM
All deaths	9895	10,080
All female deaths within age 15‒49 years	1582	1643
Total pregnancy-related deaths	90	221
Proportion of pregnancy-related deaths	0.0569	0.1345

*Source*: Mizoguchi and West [24]

The census column only includes census deaths that could be validated by INCAM

#### Mozambique DHS 2003 and 2011

**Fig. 5:**
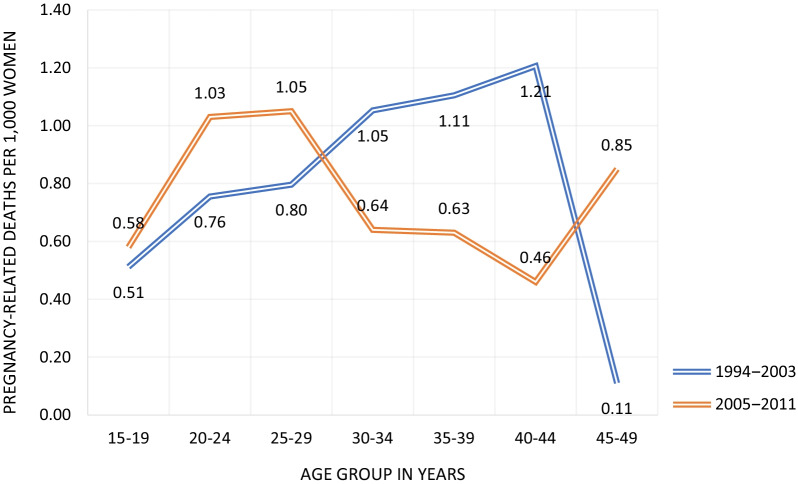
Maternal mortality rate by age groups estimated by sisterhood method, Mozambique DHS 2003 and 2011. *Data Source*: MISAU et al. [22, 23]

DHS uses the indirect sisterhood method to collect information on pregnancy-related mortality. Theoretically, the sisterhood method is equivalent to the direct method used in a census, though the empirical relationship between the two methods has not been well-established. Mozambique 2011 DHS survey collected information on pregnancy-related deaths from a nationally representative sample [23]. The reference period of INCAM of June 2006–June 2007 is almost in the middle of the DHS 2011’s reference period of July 2004–July 2011. As a result, the estimates of the two sources should be comparable. The ratio of estimates of pregnancy-related deaths to all female deaths age 15‒49 of 13.8 from DHS 2011 is nearly identical to that from INCAM. Although with a much earlier reference period of March 1997–March 2004, DHS 2003 also estimated a similar ratio of estimates of pregnancy-related deaths to all female deaths age 15‒49 of 14.3 [22]. The consistency between those three estimates suggests slow progress in reducing maternal mortality in the country. The pregnancy-related mortality rate by age groups estimated by sisterhood methods are shown in Fig. 5.

Table 4 summarizes the estimates obtained from the three different sources of pregnancy related and/or maternal mortality data for Mozambique.

**Table 4:** Comparison of estimates of pregnancy-related and maternal mortality ratios from the 2007 post-census mortality survey, 2003 and 2011 demographic and health surveys for Mozambique

Year	Estimate (per 100,000 live births)	Measure	Source
1994‒2003	408	Pregnancy-related mortality ratio	Mozambique DHS 2003 (Sisterhood)
1997‒2003	469	Pregnancy-related mortality ratio	Mozambique DHS 2003 (Sisterhood)
2007	500	Maternal mortality ratio	Mozambique Post-census mortality survey 2007 (Census and Verbal Autopsy)
2005‒2011	443	Pregnancy-related mortality ratio	Mozambique DHS 2011 (Sisterhood)

*Source*: The DHS Program STATcompiler (https://www.statcompiler.com/en/); MISAU et al. [22], MISAU et al. [23], INE et al. [25]

### Bolivia

#### Post-census maternal mortality survey

Bolivia 2001 census asked for information on pregnancy-related deaths using September 5, 2001, as the reference date. However, the questionnaire design was flawed in several ways. First, the question was phrased as “During the year 2000, did any person who lived with you die? (Yes/No)”, deviating from the recommended reference period of the preceding year (in month). Second, it did not allow the possibility of more than one death. Third, it did not ask for the date at which the death occurred. This information might have provided a validity check as a follow-up survey found that many reported deaths actually took place in 1999 or 2001.

A post-census survey was conducted to collect more information on pregnancy-related deaths. The survey covered all the 1504 households with pregnancy-related deaths to women age 15‒49; 15% of the households with female deaths age 15‒49 years but due to causes unrelated to pregnancy; 60% of households with pregnancy-related deaths of undeclared age. The fieldwork of the follow-up survey took place in July*‒*August 2002, nearly a full year after the census [15].

The percentage of follow-up interviews that could be realized was very high at 89%. But due to the problems in the original census questionnaires, the post-census survey found that only 28% of pregnancy-related deaths to women age 15‒49 were valid. The misclassified cases include deaths of men, women of the wrong age, deaths that took place outside of the reference period, and deaths that actually did not happen at all. After applying the Generalized Growth Balance method to adjust for the underreporting in deaths and conventional Brass method to adjust births, the post-census survey suggested an MMR of 266 per 100,000 live births.

#### Bolivia DHS 2003

Bolivia 2003 DHS estimated an MMR of 229, which is close to the estimate from the 2001 census [29]. As mentioned before, the estimates of MMR from post-census mortality survey and DHS may not be directly comparable due to the difference in data collection methodologies.

Table 5 summarizes the estimates obtained from the three different sources of pregnancy related and/or maternal mortality data for Bolivia.

**Table 5:** Comparison of estimates of maternal mortality ratios from the 2002 post-census mortality survey, 2003 demographic and health survey for Bolivia

Year	Estimate (per 100,000 live births)	Measure	Source
1998‒2003	229	Maternal mortality ratio	Bolivia DHS 2003 (Sisterhood method)
2000	266	Maternal mortality ratio	Post-census maternal mortality survey (Generalized Growth Balance and Brass methods)

*Source*: Sardán et al. [29], Hakkert [15]

## Discussion

Low- and middle-income countries need options for measuring maternal mortality, as data on maternal mortality is needed in regular intervals to assess progress against global and country-specific targets. Maternal cause of death information is also crucial in enabling countries to plan programs and allocate resources. While countries are building up their civil registration systems, they need options for obtaining timely pregnancy-related or maternal mortality estimates. In this paper, we compare options for estimating pregnancy-related or maternal mortality in Bangladesh from three different data sources—the 2011 population census, the BMMS 2010 and data from Bangladesh’s SVRS 2006‒2011. We also presented findings from post-census mortality survey and DHS for Mozambique and Bolivia to provide guidance to other countries on the pros and cons of different methods for estimating MMR.

There is no gold standard against which to compare the pregnancy-related and maternal mortality estimates from these three data sources. Comparisons to international estimates from agencies such as the World Health Organization are not appropriate because they use model-based estimation with data from different sources as inputs. Despite the lack of a gold standard, we find the estimates from each country are broadly consistent with one another. These two measures, viz. PRMR and MMR, are often compared with each or used interchangeably. The sample survey estimate of 194 maternal deaths per 100,000 live births (for the period 2010‒2011) is quite similar to the BMMS 2010 estimates from the household method (with and without verbal autopsy) and the SVRS pregnancy-related mortality estimates for 2010‒2011. The SVRS estimates are close to the BMMS 2010 estimates from sisterhood but are somewhat higher than the estimates from the household method.

The marginal costs of adding a question about pregnancy-related mortality into an already planned census is likely to be quite small. This is one key advantage of this methodology. In addition, when relevant questions are included in a full census, the national coverage implies a larger number of cases than regular surveys. That increases the accuracy in estimation, which is an issue for other methods, and makes it possible to estimate pregnancy related mortality both by subregion and subgroup. There is also the possibility that a census could be followed by a post-census verbal autopsy to obtain maternal mortality estimates, as was done after the 2007 Mozambique census [25]. However, experience from Bolivia demonstrates that the follow-up mortality survey has to take place within 6 months after the census—the 2002 post-census maternal mortality survey took place nearly a year after the 2001 Bolivia population census and only 28% of the pregnancy-related deaths identified in census were found to be valid [15]. A disadvantage of the census method is that it provides estimates only every 10 years or so. The Bangladesh 2011 population census provided estimates for a period from about October 2010‒October 2011. In the year 2020, these estimates may already be outdated and can’t be used for monitoring progress over periods of less than 10 years. Quality of data and reporting in census reports also require attention—for example, by definition, PRMR was calculated but reported in the census report for Bangladesh as MMR or MMRate [6]. In addition, evaluation and adjustment of data on births, deaths, and attribution of deaths as being maternal should be an essential part of the exercise for any country that uses census for estimating maternal mortality [31].

The direct sisterhood method has been used in household surveys such as the DHS and the BMMS. In this method, a woman is asked about the survival status of all her adult sisters. For sisters who passed away, questions are asked about the timing of death and whether she was pregnant, died during childbirth or died within two months of pregnancy [28]. This method provides estimates for several time periods before the survey. For the BMMS 2010 the time periods were 1996–2000, 2001–2005, 2006–2010 and 2008–2010. Though by asking one respondent about all of her sisters, the sample size expands [21], the samples still tend to be small and sample errors could potentially be large [17]. The BMMS 2010 overcame this challenge by sampling 175,000 households (for comparative purposes, 19,457 households were sampled for the 2017 Bangladesh Demographic and Health Survey). The BMMS 2010 also provided estimates of pregnancy-related mortality by asking household heads about deaths since October 2006. A follow-up verbal autopsy was conducted for households reporting a death to women aged 13–49 years. The follow-up verbal autopsy allowed for the estimation of maternal mortality for the period 2007–2010.

Though surveys such as the BMMS 2010 are a rich source of information and often considered as adopting “gold standard” methodologies for tracking progress in maternal and child health indicators [16], the cost of sampling such a large number of households may be too high. Despite the large sample of the BMMS 2010, maternal deaths are statistically such a rare events that even a sample of 175,000 households was unable to detect the statistical significance of an apparently substantial decline in PRMR over a 10-year period—five-year PRMR estimated by sisterhood method for 1998 was 382 (95% CI 328, 438) and for 2008 was 301 (95% CI 256, 346), and hence the 21% reduction was not statistically significant [26]. A similar issue was observed in the BMMS 2001, where a 22% decline in PRMR during 1988‒1999 found to be not statistically significant due to wide confidence intervals [3, 18]. A systematic comparison of different approaches to estimate maternal mortality using the BMMS 2001 concluded that surveys do not have the potential to be a cost-effective strategy for routine monitoring and evaluation of maternal health [18]. In addition as maternal mortality falls in a country, larger sample sizes would be needed to provide estimates. For example, 298,284 households were interviewed for the latest round of BMMS in 2016, where a 95% confidence interval is around 23% of the assumed MMR value [27]. Experience from estimating maternal mortality from DHS highlights that wide confidence interval associated with pregnancy-related or maternal mortality estimates in most of the cases prevents one from drawing conclusions regarding change over time. Also, adding questions for sisterhood methods requires additional training and supervision in the field and adds considerable complexity to data processing [30].

SVRS is similar in concept to a civil registration system, with the exception that it does not cover an entire country. The Bangladesh SVRS is designed to be national in scope and vital events are captured in a dual reporting system. The Bangladesh SVRS obtains information on cause of maternal death which is reported by lay individuals. Two major issues for Bangladesh SVRS are, however, data quality and estimation process of sampling error. First, SVRS collects cause of death data by local registrar or officials from the district/upazila statistical office under the dual recording system. Documentation on data quality, field implementation, and data collection training assessment for SVRS are not publicly available.

Considering the pros and cons of the aforementioned methods for estimating maternal mortality, SVRS has the potential to provide routine monitoring information on maternal mortality. While SVRS are not yet widespread in low- and middle-income countries, there is growing interest in their use in more countries. For example, Mozambique launched a SVRS in 2017 and this system has been producing nationally and provincially representative vital statistics for several years [5], while Sierra Leone is planning to produce estimates after its first year of data collection.

Over time Bangladesh could consider SVRS expansion to cover the whole nation, as this option would line with the country’s commitment to a strong civil registration system [13]. In such a case the census method could be used to calibrate the sample frame and adjust for any bias due to under- or over-representation of certain groups in the SVRS. Combining brief information from a large census and the detailed information from a relatively small SVRS may also offer the opportunity to generate disaggregated estimates (e.g., by division, even district). This approach is suitable for other countries who are struggling with maternal mortality estimation in regular intervals, and working toward a national civil registration system.

## Conclusion and way forward

Overall all these data sources presented in this paper have provided valuable information on maternal mortality in Bangladesh, Mozambique and Bolivia. Based on the findings and review of methodologies presented in this paper, we highlight the following options for collecting reliable and contemporaneous pregnancy-related and/or maternal mortality estimates in a resource-constrained setting while progressing toward a complete civil registration system:

- Looking forward, the SVRS is the option that could propel the low- and lower-middle income countries toward having a complete civil registration system. SVRS can provide routine monitoring information on PRMR up to sub-national levels. However, quality of data collected under SVRS needs to be monitored closely and indicator calculation methods need to be transparent and robust enough to draw conclusions regarding PRMR change over time.
- For SVRS, post-census mortality survey, and household sample surveys, causes of death collected should be aligned with the ICD-11 to increase precision and comparability to other settings [40]. A recommendation would be to consider using clinical review or automated methods, as suggested in the WHO’s newly developed 2016 verbal autopsy standards, which is suitable for routine use [39].
- Develop and implement a multi-year, comprehensive capacity-building plan for improving skills, and competence of the National Statistical Office in routine mortality data collection and analysis in close collaboration with the Ministry of Health.
- Establish an independent Indicator Reference Group [2], by forming partnerships among agencies within the government agencies, research organizations, development partners, NGOs, and professional bodies, to facilitate maternal mortality data analysis, triangulation and support for evidence-based decision making.

## Supplementary Information

**Additional file 1**. Supplement 1. Detailed description of the methods for measuring maternal mortality.

## Data Availability

The data used in this paper are all from publicly available sources and reports from the Demographic and Health Surveys, Dataverse, census reports and IPUMS.

## Abbreviations

BMMS: Bangladesh Maternal Mortality Survey
DHS: Demographic and Health Survey
HIS: Health Information System
INCAM: Mozambique Inquerito Sobre Causas de Mortalidade
MDG: Millennium Development Goal
MMEIG: Maternal Mortality Estimation Inter-agency Group
MMR: Maternal Mortality Ratio
MMRate: Maternal Mortality Rate
PRMR: Pregnancy-related Mortality Ratio
SVRS: Sample Vital Registration System

## References

[1] Ahmed S, Li Q, Scrafford C, Pullum TW. An assessment of DHS maternal mortality data and estimates. DHS Methodological Reports No. 13. Rockville: ICF International; 2014.

[2] Ahsan KZ, Tahsina T, Iqbal A, Ali NB, Chowdhury SK, Huda TM, Arifeen SE (2017). Production and use of estimates for monitoring progress in the health sector: the case of Bangladesh. Glob Health Action.

[3] Alam N, Townend J (2014). The neighbourhood method for measuring differences in maternal mortality, infant mortality and other rare demographic events. PLoS ONE.

[4] Alkema L, Chou D, Hogan D, Zhang S, Moller AB, Gemmill A, Fat DM, Boerma T, Temmerman M, Mathers C, Say L (2016). Global, regional, and national levels and trends in maternal mortality between 1990 and 2015, with scenario-based projections to 2030: a systematic analysis by the UN Maternal Mortality Estimation Inter-Agency Group. Lancet.

[5] Amouzou A, Kante A, Macicame I, Antonio A, Gudo E, Duce P, Black RE (2020). National sample vital registration system: a sustainable platform for COVID-19 and other infectious diseases surveillance in low and middle-income countries. J Glob Health.

[6] Bangladesh Bureau of Statistics (BBS). Population and housing census 2011: socio-economic and demographic report. National series, vol. 4. Dhaka: Statistics and Informatics Division, Ministry of Planning; 2012.

[7] Bangladesh Bureau of Statistics (BBS) (2013). Report on sample vital registration system 2011.

[8] Bangladesh Bureau of Statistics (BBS). Population and housing census 2011: analytical report. National series, vol. 1. Dhaka: Statistics and Informatics Division, Ministry of Planning; 2015.

[9] Chandrasekaran C, Deming WE (1949). On a method of estimating birth and death rates and the extent of registration. J Am Stat Assoc.

[10] Chandrasekaran C, Deming WE. On the correlation bias in the application of Chandra-Deming method for estimating vital events. Working paper no. 2. Cairo: Cairo Demographic Centre; 1981.

[11] Donati S, Maraschini A, Buoncristiano M (2016). Methods to estimate maternal mortality: a global perspective. J Epidemiol Community Health.

[12] EPMM Working Group (2015). Strategies toward ending preventable maternal mortality (EPMM).

[13] Government of Bangladesh (GOB) (2013). Report of the comprehensive assessment and strategic action plan on civil registration and vital statistics (CRVS) system in Bangladesh.

[14] Graham W, Brass W, Snow R (1989). Estimating maternal mortality: the sisterhood method. Stud Fam Plan.

[15] Hakkert R (2011). Follow-up surveys for census estimates of maternal mortality: experiences from Bolivia and Mozambique. J Population Res.

[16] Hancioglu A, Arnold F (2013). Measuring coverage in MNCH: tracking progress in health for women and children using DHS and MICS household surveys. PLoS Med.

[17] Hill K, Stanton C, Gupta N. Measuring maternal mortality from a census: guidelines for potential users. Measure evaluation manual series, No. 4. Chapel Hill: MEASURE Evaluation; 2001.

[18] Hill K, Arifeen SE, Koenig M, Al-Sabir A, Jamil K, Raggers H (2006). How should we measure maternal mortality in the developing world? A comparison of household deaths and sibling history approaches. Bull World Health Org.

[19] Instituto Nacional de Estadistica (INE). Encuesta Postcensal de Mortalidad Materna: Resultados finales para el an˜o 2000. [Post-census Investigation of Maternal Mortality: Final Results for the Year 2000]. La Paz: INE; 2003.

[20] Loureiro JD. The 2007 Mozambique’s census and its organization. Paper presented in seminar on recent experiences in Population and Housing Census, New York; 2008.

[21] Mgawadere F, Kana T, van den Broek N (2017). Measuring maternal mortality: a systematic review of methods used to obtain estimates of the maternal mortality ratio (MMR) in low- and middle-income countries [published correction appears in Br Med Bull. 2017 Jun 1;122(1):1]. Br Med Bull.

[22] Ministry of Health (MISAU), National Statistics Institute (INE), ORC Macro. Mozambique demographic and health survey 2003. Calverton: MISAU, INE and ORC Macro; 2005.

[23] Ministry of Health (MISAU), National Statistics Institute (INE), ICF International (ICFI). Mozambique demographic and health survey 2011. Calverton: MISAU, INE and ICFI; 2013.

[24] Mizoguchi N, West L. How well do censuses capture pregnancy-related deaths? A case study from Mozambique. Paper presented in IUSSP 28th international population conference, Cape Town, South Africa; 2017.

[25] Mozambique National Institute of Statistics (INE), U.S. Census Bureau, MEASURE Evaluation, U.S. Centers for Disease Control and Prevention. Mortality in Mozambique: results from a 2007–2008 post-census mortality survey. Chapel Hill: MEASURE Evaluation; 2012.

[26] National Institute of Population Research and Training (NIPORT), MEASURE Evaluation, icddr,b. Bangladesh maternal mortality and health care survey 2010. Dhaka: NIPORT, MEASURE Evaluation, icddr,b; 2012.

[27] National Institute of Population Research and Training (NIPORT), International Centre for Diarrhoeal Disease Research, Bangladesh (icddr,b), and MEASURE Evaluation. Bangladesh maternal mortality and health care survey 2016: final report. Dhaka: NIPORT, icddr,b, MEASURE Evaluation; 2019.

[28] Rutenberg N, Sullivan J (1991). Direct and indirect estimation of maternal mortality from the sisterhood method.

[29] Sardán MG, Ochoa LH, Guerra WC. Bolivia National Demographic and Health Survey 2003. Calverton: Instituto Nacional de Estadística/Bolivia and ORC Macro; 2004.

[30] Stanton C, Abderrahim N, Hill K. DHS maternal mortality indicators: an assessment of data quality and implications for data use. DHS analytical reports no. 4. Calverton: Macro International Inc; 1997.

[31] Stanton C, Hobcraft J, Hill K, Kodjogbe N, Mapeta WT, Munene F, Naghavi M, Rabeza V, Sisouphanthong B, Campbell O (2001). Every death counts: measurement of maternal mortality via a census. Bull World Health Org.

[32] Sullivan TR, Hirst JE (2011). Reducing maternal mortality: a review of progress and evidence-based strategies to achieve millennium development goal 5. Health Care Women Int.

[33] United Nations Principles and Recommendations for Population and Housing Censuses, Revision 2. Statistical papers series M No. 67/Rev.2. New York: Department of Economic and Social Affairs, Statistics Division; 2008.

[34] United Nations. ‘All people benefit’ when maternal health care is improved: UN Secretary-General’s message marking the fifteenth anniversary of population and development conference (ICPD). 2009. https://www.un.org/press/en/2009/sgsm12539.doc.htm.

[35] World Bank and World Health Organization (WHO) (2014). Global civil registration and vital statistics: scaling up investment plan 2015–2024.

[36] World Health Organization (WHO). International classification of diseases, 10th Revision. Geneva: WHO; 2004. http://www.who.int/classifications/icd/en/.

[37] World Health Organization (WHO) (2010). Trends in maternal mortality: 1990–2008: estimates developed by WHO, UNICEF, UNFPA, World Bank Group and the United Nations Population Division.

[38] World Health Organization (WHO) (2015). Trends in maternal mortality: 1990–2015: estimates by WHO, UNICEF, UNFPA, World Bank Group and the United Nations Population Division.

[39] World Health Organization (WHO). Verbal autopsy standards: the 2016 WHO verbal autopsy instrument, v1.5. Geneva: WHO; 2017. https://www.who.int/healthinfo/statistics/verbalautopsystandards/en/.

[40] World Health Organization (WHO). International classification of diseases, 11th revision. Geneva: WHO; 2018. http://www.who.int/classifications/icd/en/.

