# Supplementary material for: A comparison of approaches to measuring maternal mortality in Bangladesh, Mozambique, and Bolivia
Source: Popul Health Metr. 2022 Jan 15;20:5. doi: 10.1186/s12963-022-00281-8 (PMC8760829; doi:10.1186/s12963-022-00281-8)
Supplement: Supplementary file 1 — Additional file 1. Supplement 1. Detailed description of the methods for measuring maternal mortality. [file 12963_2022_281_MOESM1_ESM.docx]

# **Additional file 1. Detailed Description of the methods for measuring maternal mortality**

Multiple approaches to measuring maternal mortality are in practice, such as using data from civil registration systems, sample vital registration systems, national population censuses, household surveys, and prospective surveillance of deaths of reproductive-aged women (WHO 2013). In the following, the methods for measuring maternal mortality highlighted in the paper are discussed, along with the advantages and disadvantages of each data source/method.

## ***Census***

Population censuses are conducted in almost every country in the world in regular intervals, usually once every 10 years, systematically collecting information about the population for resource allocation and planning purposes. Following the recommendation of the United Nations Principles and Recommendations for Population and Housing Censuses, a national census can be used to obtain estimates of maternal mortality with the addition of several additional follow-up questions to the census schedule for countries lacking a complete vital registration system (WHO, 2013; Mgawadere, Kana & van den Broek, 2017). The World Health Organization recommended the following Mortality Questions box.:

1. Has any member of this household died in the last 12 months?
2. If yes, record the following information about each deceased person:

| Name | Sex | Age at death (in completed years) | Was the death due to an accident, violence, homicide, or suicide? | Maternal Mortality Questions:  If the deceased was female aged 15–49 at the time of death, was she: | | |
| --- | --- | --- | --- | --- | --- | --- |
|  |  |  |  | Pregnant? | Giving birth? | Within 6 weeks of the end of a pregnancy or childbirth? |
|  |  |  | Yes No | Yes No | Yes No | Yes No |
|  |  |  | Yes No | Yes No | Yes No | Yes No |
|  |  |  | Yes No | Yes No | Yes No | Yes No |

The national population census allows the identification of deaths in a household in a short reference period (1–2 years) and provides recent estimates of pregnancy-related mortality ratio (PRMR). Due to its wide coverage, census produces robust PRMR estimates (theoretically covering an entire populatation) and permits subnational analyses. The estimation process involves internal data evaluation and (substantial) adjustments while using census data to estimate PRMR, which is the number of pregnancy-related deaths (multiplied by 100,000) divided by the number of live births. To calculate the PRMR, the number of live births during the reference period and the number of deaths of women of reproductive age (WRA, i.e., women age between 15 and 49 years) are needed. By using data from two consecutive censuses, births and deaths are adjusted to represent intercensal fertility and mortality, respectively, as closely as possible (Hill et al. 2009).

Regarding the adjustment of fertility, most analyses rely on the Brass P/F Ratio Method that compares lifetime fertility (P) to cumulated current fertility (F) (Brass 1975). This method has limitations, especially in countries with declining fertility rates (Moultrie & Dorrington, 2008). In addition, since no comparable proportion of pregnancy-related deaths are available for the intercensal period, an adjustment factor for all deaths is generally used in this case (Hill et al. 2009; Hill & Stanton, 2011). Apart from Bangladesh, several countries have used the national census to estimate PRMR. Studies exploring the performance of census data to estimate PRMR offered mixed results and found that the PRMR estimates from censuses were generally higher than estimates obtained from sample surveys (Dorrington et al. 2006; Hill et al. 2009; Hill & Stanton, 2011; Mgawadere, Kana & van den Broek, 2017; Hill et al., 2018).

## ***Post-census mortality survey***

Given the concerns with the quality of census data and the reliability of the PRMR estimates, several countries have conducted follow-up studies to measure pregnancy-related and maternal mortality based on the universal coverage of the national population census (Hakkert, 2011). For the post-census mortality surveys, the national census provides the sample frame. A sample of clustered enumeration areas from the census is randomly selected to be representative at the national, provincial, and urban/rural levels. The completed census form contained questions regarding deaths in the household within a pre-defined reference period (viz., the previous 12 months). Once specific clusters were chosen in each province, all households that reported a death within the cluster during the census interview were identified for a follow-up verbal autopsy (VA) interview. Trained interviewers visited the household and filled out the forms to link census data and the cause of deaths, which are reviewed by a team of physicians trained in VA certification and coding. The physicians used the information on the forms, their medical expertise, and ICD-10 certification and coding guidelines to complete a death certificate indicating the immediate and underlying causes of each death reported during the reference period (Singh & Hart, 2015). In practice, only a small proportion of all the households in the country is selected for a post-census mortality survey. For example, approximately a five-percent sample of all households in Mozambique was selected in 2007 (Hemed et al. 2009).

This complementary approach makes the best possible use of both census information and survey data to obtain a complete set of potential maternal deaths with more detail and greater accuracy to estimate the PRMR and maternal mortality ratio (MMR). In order to measure maternal mortality accurately, this approach allows to break down the causes of death data into actual direct causes of maternal mortality and different types of non-maternal causes of death. In addition, post-census mortality surveys can also identify deaths among WRA that the census incorrectly classified not related to pregnancy (Hakkert, 2011). However, the post-census mortality surveys require considerable care in designing and implementing to get high-quality results. Based of a review of post-census mortality surveys in Bolivia, Mozambique, and North Korea, Hakkert (2011) highlighted the following lessons learned while carrying out a post-census mortality survey: a) such surveys are expensive because deaths in the sampled clusters have to be investigated wherever they occurred, even in hard-to-reach areas; b) formulation of the census questions needs to precisely follow the recommendations by the United Nations (2008) to measure both maternal and pregnancy-related mortality through post-census survey, since the problems with census data may result in inaccurate measurement and/or unexpectedly expand the required sample size of the follow-up survey; c) execution of the follow-up survey needs to be within 6 months after the census to facilitate locating the selected households (i.e., that reported the death of a woman of reproductive age during census) and reduce the risks of recall bias in regard to the relevant events; and d) ensure appropriate considerations to the possible errors/classification issues related to survey aim or intrinsic to the census data.

## ***Household survey***

In the absence of complete vital registration with good attribution of causes of deaths in many countries, household surveys are the most commonly employed methods for estimation of maternal mortality (Ahmed et al., 2014). In the household surveys, respondents are sampled in such a way that the survey sample represents the population (women age 15-49) of the country, and their fertility and mortality information is collected. The valid estimation of maternal mortality requires accurate and complete reporting of the events (deaths due to maternal causes) among at-risk women (age 15-49) during a reference calendar period, and the household surveys employ several approaches to estimate nationally representative PRMR/MMR in a country. The methods of maternal mortality estimation currently used in the household sample surveys are as follows:

### Sisterhood method

This method consists of obtaining information by interviewing a representative sample of female respondents of reproductive age about the survival of all their adult sisters in order to determine the number of ever-married sisters, whether they are alive or dead, and how many of them died during pregnancy, delivery or within two months of pregnancy. This method assumes that the reported sisters constitute a representative sample of the country’s population and that it is possible to calculate unbiased estimates of maternal mortality indicators from these data. In this approach, a respondent is asked to provide the birth history of her mother, including the current age of all living siblings and the age at death and years since death for all deceased siblings. These data allow deaths and births to be placed in calendar time and, therefore, permit the calculation of sex and age-specific death rates for reference periods (Mgawadere, Kana & van den Broek, 2017).

The sisterhood method also relies on the assumption that the sampled respondent can report accurately on the age of living sisters and the age at death and years since death for dead sisters. This is a big assumption, especially when the respondents are the youngest in the family and may not remember or know about siblings that were born and died either before they were born or when they were too young to remember. In addition, being aware of the pregnancy status and timing of siblings requires a significant recall. A previous assessment of the sisterhood method used by DHS reported relative errors in maternal mortality estimates of 15% across 13 countries when the recall period was 0 to 6 years before the survey (Ahmed et al., 2014; Stanton et al., 2000). Another potential source of bias in this method results from the assumption that the risk of mortality among sisters is independent, i.e., the risk of a sister dying is not related to the risk of another sister in that family dying. If mortality risk is related, then sisters with a higher risk of dying are less likely to be available to respond and thus underestimate the mortality level (Ahmed et al., 2014).

The underreporting of sisters, as well as deaths of sisters, is also common using this method. As mentioned above, the age of the respondent will affect the accuracy of the estimate. Recent evaluations of this approach noted substantial omission of siblings in a respondent’s history and death reporting, but little difference in the amount of missing data the between the standard tools used for the direct sisterhood method and a more detailed instrument (Hellerringer et al. 2014a; Hellerringer et al. 2014b).

### Direct household method of estimation

While the majority of the countries with DHS surveys rely on the direct sisterhood method of measuring maternal mortality, several countries have implemented the direct household method (Ahmed et al., 2014). It incorporates questions about deaths that occurred in the sampled household, regardless of sibling status, for a predefined recall period prior to the survey. For deaths among WRA, there are further questions related to the timing of death to determine if the death was pregnancy-related. The female mortality data are followed up with verbal autopsies where the family members or other people with knowledge about the death could be asked to describe the situation surrounding the dead relatives to ascertain the maternal deaths (Mgawadere et al., 2017). These surveys require a large sample size to obtain statistically significant findings for rare events such as maternal mortality. For this reason, most countries rely on the sisterhood method as part of a DHS survey or another small-scale sample survey (Ahmed et al., 2014).

Very few studies have compared the PRMR estimates between the direct household method and the sisterhood method. Results from Bangladesh indicated that pregnancy-related deaths estimated with the household and sisterhood methods were similar (i.e., not significantly different), while estimates of maternal deaths from the household survey were about 15% lower. However, results from Afghanistan found the PRMR from the direct household method to be 39% higher than the estimate from the sisterhood method (Ahmed et al., 2014).

## ***Sample vital registration system (SVRS)***

An SVRS uses a national random sample of communities to continuously track population and demographic events to measure multiple indicators, including mortality. It monitors births, deaths, and migrations in a sample of individuals, households, and/or residential units in a specific area and is able to provide accurate estimates for the population during the intercensal period.

The size and coverage of SRVS vary greatly across countries—countries such as China and India have implemented SVRS nationally, whereas other countries (such as Zambia and Tanzania) implemented sample vital registration with verbal autopsy (SAVVY) to produce location-specific estimates of vital events with causes of death (Ye et al. 2012; Curtis, Mswia & Weaver, 2015). The Bangladesh SRVS was initiated in the 1980s in 103 communities located across the country and progressively increased to 2012 communities, covering about 700,000 people. To ensure data quality, internal validation and close supervision of data collection is done under SVRS. Data are collected by the local registrars, and the quality of the data is checked by supervisors. Filled-in schedules are then sent to headquarters on a monthly basis. Rechecking is done by Regional Statistical Officers and other officers and staff members. In the first recording method, a local registrar reports on events as they occur. The local registrar collects data on births and deaths (and other events) on an ongoing basis and sends the data to the headquarter office on a monthly basis. In the second recording method officials from the district/upazila statistical office employ the same schedules (i.e., reporting tools) to retrospectively capture events on a quarterly basis. Data from the two recording methods are compared using pre-determined matching criteria. When there are partially matched or non-matched events, field visits are conducted to reconcile the information (BBS 2020).

SVRS serves as an alternative to exhaustive monitoring of the entire country’s population, as in civil registration and vital statistics (CRVS) systems (Amouzou et al. 2020). When designed and implemented well, SVRS can produce reliable estimates for the target population. A common concern for SVRS is its sample size, which is likely to be too small to detect a short-term change in mortality levels, particularly for rare events such as maternal mortality. Also, the process of assigning the cause of death does not use the WHO-recommended global standard tool on Verbal Autopsy, which reduces the validity of the cause of death data from SVRS. For Bangladesh, over-representation of the urban sample and a lack of independence in the dual reporting systems in SVRS have been highlighted as the major shortcomings of this data source.

## ***Other sources***

Alternative sources of data for measuring maternal mortality include Reproductive-Age Mortality Studies (RAMOS) and Health and Demographic Surveillance System (HDSS) (Dorrington & Bradshaw, 2011). Specialized studies such as RAMOS have been identified as a relatively robust method, which uses multiple data sources, including health facility records, to estimate the MMR in countries without an adequate vital registration system. The approach involves retrospective or prospective identification and investigating the causes of all deaths among WRA in a defined area/population by using multiple sources of data such as existing records (CRVS and health facility data), census, surveys, and surveillance. RAMOS is conducted in two phases. The first phase involves identification of all deaths among WRA in a population, and in the second phase, all deaths are investigated (using verbal autopsy, health facility reports, or medical record reviews death certificates with medical cause and interviews with household members and relatives) to ascertain if there are pregnancy-related or maternal deaths. Health facility reports and medical records considered for RAMOS include admission and discharge books, death certificate books, death registers, mortuary logbooks, and individual case notes when necessary. Other relevant data include the number of births from the most recent DHS and the routine Health Information System (HIS), and deaths that occurred in the community were identified by local key informants, traditional birth attendants, and community workers (Mgawadere, Kana & van den Broek, 2017).

Available RAMOS studies indicate that, based on the strengths and feasibility of application, RAMOS provides reliable, most complete, and contemporaneous estimates of MMR in the absence of a CRVS. Studies from Malawi, Sudan, Jordan, and Ghana showed that RAMOS identified more maternal deaths than obtained via any one of the existing reporting mechanisms alone (e.g., HIS or facility death reports). However, this approach is difficult to implement in the absence of a reasonably complete initial list of deaths. RAMOS can also be expensive and time-consuming when conducted on a larger scale. Similarly, while HDSS is considered as the gold standard for producing high-quality data mortality and causes of death (Alam et al. 2017), the coverage of HDSS is very specific and can be very expensive when implemented at scale.

# **Additional file 1: Table S1. Summary of advantages and disadvantages of different data sources for maternal mortality estimation**

| **Data source** | **Advantage** | **Disadvantage** |
| --- | --- | --- |
| Census | Involves well-developed, formal evaluation methods | Identifies pregnancy-related deaths (not maternal deaths) |
|  | No sampling errors (because all women are covered) | Census misses deaths in single-person households |
|  | Allows a more detailed breakdown of the results, including trend analysis and estimate differentials by socioeconomic and geographic variables | Needs careful evaluation of the basic data, and results must be adjusted for completeness of death and birth statistics, and population structures produce reliable estimates |
|  | Allows identification of death in a relatively short reference period (1–2 years) | Conducted in long intervals (generally in every 10 years), which limits the monitoring of maternal mortality |
|  | Relatively low marginal cost |  |
| Post-census mortality survey | Cost of death identification is absorbed by census and can potentially leverage donor/sectoral financial support | Can only be conducted with census, which limits the monitoring of maternal mortality |
|  | May increase targeted sample size easily by adjusting sampling fraction | Due to (relatively) poor quality census data, many out-of-frame deaths identified in census by post-census survey |
|  | 12 month recall period is standard on most censuses, accepted as ‘reasonable’ for verbal autopsy | Requires long lead time for planning (estimated 15+ months before census) |
|  | Ability to calculate cause-specific mortality fractions at subnational level | Requires link back to census data to calculate rates and ratios |
|  | Data quality can be checked by comparing mortality information with the census mortality statistics | Requires at least two visits to household, may result in loss of households that cannot be re-identified during follow up |
| Household survey (sisterhood method) | Low cost | Identifies pregnancy-related deaths (not maternal deaths) |
|  | Reduces the need for large sample sizes because there may be more than one respondent per household and more than one sister per respondent | Provides retrospective rather than actual maternal mortality estimate (over 5–10 years prior to survey), therefore, cannot be used for evaluating the impact of an intervention |
|  | Allows deaths and births to be placed in calendar time to permit the calculation of sex and age-specific death rates for reference periods | Difficult to get additional information about deaths (risk factors, timing, etc.) as sibling may not have such details, and abortion-related deaths are often not captured |
|  | The approach also allows for the calculation of rates/ratios for the reference period of interest and monitor trends. | Less appropriate in settings with significant migration and population movement |
|  |  | Recent fertility decline implies fewer female siblings and results in smaller sample sizes and elevated sampling errors |
| Household survey (direct estimation) | Fieldwork is logistically relatively simple, can be planned for in 4–6 months | Sample size of deaths relatively small even with large sample size |
|  | 3-year recall period followed by verbal autopsy allows cost-effective, reliable identification of maternal deaths | Produces estimates with wide confidence intervals, hence can only detect large relative changes in MMR |
|  | Can also collect information on causes time, place, health care seeking behavior prior to death | Costly |
| SVRS | Allows continuous data collection once established | Expansion of SVRS is costly |
|  | With most deaths occurring at the community level, it is an ide­al platform for generating representative estimates | Deviation from standard verbal autopsy tool may reduce the reliability of mortality estimates |
|  | Complements and reinforces other existing data collection systems | Although it provides ongoing data, sample size per year is likely to be too small to detect short term change. |

Sources: Stanton et al. 2000; Hill & Stanton 2010; Mgawadere, Kana & van den Broek 2017; Curtis et al. 2015; WHO 2019

# **References**

Ahmed S, Li Q, Scrafford C, Pullum TW. An Assessment of DHS Maternal Mortality Data and Estimates. DHS Methodological Reports No. 13. Rockville, Maryland, USA: ICF International. 2014.

Alam N, Ali T, Razzaque A, Rahman M, Zahirul Haq M, Saha SK, Ahmed A, Sarder AM, Moinuddin Haider M, Yunus M, Nahar Q. Health and demographic surveillance system (HDSS) in Matlab, Bangladesh. International Journal of Epidemiology. 2017; 46(3):809-816.

Amouzou A, Kante A, Macicame I, Antonio A, Gudo E, Duce P, Black RE. National Sample Vital Registration System: A sustainable platform for COVID-19 and other infectious diseases surveillance in low and middle-income countries. Journal of global health. 2020; 10(2): 020368.

Bangladesh Bureau of Statistics (BBS). Report on sample vital registration system 2019. Dhaka: Statistics and Informatics Division, Ministry of Planning. 2020.

Brass W. Methods for estimating fertility and mortality from limited and defective data. Methods for estimating fertility and mortality from limited and defective data. Chapel Hill, NC: International Program of Laboratories for Population Statistics. 1975.

Curtis SL, Mswia RG, Weaver EH. Measuring maternal mortality: Three case studies using verbal autopsy with different platforms. PloS One. 2015; 10(8):e0135062.

Dorrington RE, Timæus IM, Gregson S. Adult mortality in Southern Africa using deaths reported by households: Some methodological issues and results. Paper presented at the Annual conference of the Population Association of America. Los Angeles, 30 March-1 April 2006.

Helleringer S, Pison G, Kanté AM, Duthé G, Andro A. Reporting errors in siblings’ survival histories and their impact on adult mortality estimates: results from a record linkage study in Senegal. Demography. 2014; 51(2): 387-411.

Helleringer S, Pison G, Masquelier B, Kanté AM, Douillot L, Duthé G, Sokhna C, Delaunay V. Improving the quality of adult mortality data collected in demographic surveys: validation study of a new siblings' survival questionnaire in Niakhar, Senegal. PLoS medicine. 2014; 11(5): e1001652.

Hemed Y, Mbofana F, Mazive E, Mswia R, Cummings S, Young P, West L. Using a Post-Census Survey to Measure Maternal Mortality. Paper presented at: XXVI IUSSP International Population Conference. Marrakech, 27 September-2 October 2009.

Hill K, Queiroz BL, Wong L, Plata J, Popolo FD, Rosales J, Stanton C. Estimating pregnancy-related mortality from census data: experience in Latin America. Bulletin of the World Health Organization. 2009; 87:288-95.

Hill K, Stanton C. Measuring maternal mortality through the census: rapier or bludgeon?. Journal of Population Research. 2011; 28(1):31-47.

Hill K, Johnson P, Singh K, Amuzu-Pharin A, Kharki Y. Using census data to measure maternal mortality: A review of recent experience. Demographic research. 2018; 39:337.

Mgawadere F, Kana T, van den Broek N. Measuring maternal mortality: a systematic review of methods used to obtain estimates of the maternal mortality ratio (MMR) in low-and middle-income countries. British medical bulletin. 2017;121(1):121-34.

Moultrie TA, Dorrington R. Sources of error and bias in methods of fertility estimation contingent on the P/F ratio in a time of declining fertility and rising mortality. Demographic Research. 2008; 19:1635-62.

Singh K, Hart L. A Guide on Conducting a Post-Census Verbal Autopsy to Estimate Maternal Mortality. Chapel Hill, NC: MEASURE Evaluation. 2015.

United Nations. Principles and recommendations for population and housing censuses. Report Series M, No. 67, Revision 2. New York: Statistics Division, Department of Economic and Social Affairs. 2008.

World Health Organization (WHO). Trends in maternal mortality 2000 to 2017: estimates by WHO, UNICEF, UNFPA, World Bank Group and the United Nations Population Division. Geneva: WHO. 2019.

Ye Y, Wamukoya M, Ezeh A, Emina JB, Sankoh O. Health and demographic surveillance systems: a step towards full civil registration and vital statistics system in sub-Sahara Africa? BMC Public Health. 2012; 12(1): 741.
